# Hydration Knowledge, Water Consumption, and Attitudes Toward Drinking Water Quality Among Adults in Romania: A Cross-Sectional Study

**DOI:** 10.3390/nu18030419

**Published:** 2026-01-27

**Authors:** Corina Dalia Toderescu, Melania Munteanu, Laura Ioana Bondar, Brigitte Osser, Roland Fazakas, Gyongyi Osser, Iosif Ilia, Ionuț Daniel Răducan, Maria Alina Andresz, Svetlana Trifunschi

**Affiliations:** 1 Faculty of Pharmacy, “Vasile Goldiș” Western University of Arad, 310045 Arad, Romania; toderescu.corina@uvvg.ro (C.D.T.); munteanu.melania@uvvg.ro (M.M.); trifunschi.svetlana@uvvg.ro (S.T.); 2 Doctoral School of Biomedical Sciences, University of Oradea, 410087 Oradea, Romania; 3 Department of Biology and Life Sciences, Faculty of Medicine, “Vasile Goldiș” Western University of Arad, 310025 Arad, Romania; fazakas.roland@uvvg.ro; 4 Faculty of Physical Education and Sport, “Aurel Vlaicu” University of Arad, 310130 Arad, Romania; gyongyi.osser@uav.ro (G.O.); iosif.ilia@uav.ro (I.I.); 5 Multidisciplinary Doctoral School, “Vasile Goldiș” Western University of Arad, 310025 Arad, Romania; 6 VID Concept Research, 077055 Bucharest, Romania; daniel.raducan@vidconcept.com; 7 The County Emergency Clinical Hospital of Ilfov, 022104 Bucharest, Romania; labrim@spitalulilfov.ro

**Keywords:** bottled water, drinking water, health knowledge, attitudes, practice, hydration, tap water, water consumption

## Abstract

**Background/Objectives**: Adequate hydration is essential for health; however, water consumption behaviors are influenced not only by physiological needs but also by hydration knowledge and perceptions of drinking water quality. Empirical evidence examining these factors in Eastern European populations remains limited. This study aimed to assess hydration knowledge, water consumption patterns, and attitudes toward drinking water quality among adults in Romania, and to examine their associations with daily water intake and water source preferences. **Methods**: A cross-sectional study was conducted between November 2024 and November 2025 among adults residing in Romania. Data were collected from 165 participants using an anonymous, self-developed, paper-based questionnaire administered in person to adult patients attending routine visits in four primary care clinics in Arad, Romania, using a convenience sampling approach. The questionnaire assessed sociodemographic characteristics, hydration knowledge, water consumption behaviors, and attitudes toward drinking water quality. Descriptive statistics, chi-square tests, correlation analyses, and multivariable linear and logistic regression models were applied to identify factors associated with daily water intake, adequate hydration (≥2 L/day), and bottled water consumption. **Results**: Hydration knowledge was moderate overall and was significantly associated with education level and gender. Higher hydration knowledge was positively correlated with daily water intake (r = 0.21, *p* = 0.006) and was independently associated with higher intake and adequate hydration (OR = 1.28, 95% CI: 1.10–1.49; *p* = 0.002). Greater trust in tap water was also positively associated with daily intake (r = 0.27, *p* = 0.001) and adequate hydration (OR = 1.31, 95% CI: 1.12–1.54; *p* < 0.001). Lower trust in tap water and stronger beliefs regarding bottled water were significant predictors of bottled water use as the primary drinking water source. Education level emerged as a consistent predictor across multiple hydration-related outcomes. **Conclusions**: Hydration knowledge and perceptions of drinking water quality are key, modifiable factors associated with water consumption behaviors. Educational strategies integrated into primary care and transparent communication regarding tap water safety may support adequate and sustainable hydration among adults.

## 1. Introduction

Adequate hydration is essential for maintaining physiological homeostasis and supporting multiple bodily functions, including thermoregulation, cardiovascular stability, renal function, and cognitive performance [1,2,3,4]. Even mild dehydration has been associated with fatigue, impaired concentration, headaches, and reduced physical performance, while chronic inadequate fluid intake may contribute to longer-term health consequences, particularly among adults and vulnerable populations [5,6,7]. Despite the recognized importance of water for health, evidence suggests that a substantial proportion of adults do not meet recommended daily fluid intake levels [8,9,10,11].

From a clinical perspective, sufficient fluid intake is particularly important for renal health, as it supports glomerular filtration, facilitates the excretion of metabolic waste products, and reduces the risk of kidney stone formation. Chronic inadequate hydration has been associated with an increased risk of nephrolithiasis, urinary tract disorders, and may contribute to the progression of chronic kidney disease, especially in vulnerable and aging populations. Given the growing global burden of kidney disease and other chronic non-communicable conditions, promoting adequate hydration represents a key, low-cost, non-pharmacological strategy for disease prevention and long-term health management [12,13,14,15,16].

Hydration behaviors are influenced by a complex interplay of biological, behavioral, and environmental factors. Beyond physiological needs, individual knowledge regarding hydration requirements, perceptions of thirst, lifestyle habits, and contextual factors such as access to safe drinking water play an important role in shaping daily water intake [17,18,19,20]. In this context, hydration knowledge can be conceptualized as a multidimensional construct encompassing both factual knowledge, such as awareness of recommended daily water intake, physiological roles of water, and common signs of dehydration, and functional knowledge, which reflects the ability to apply this information in daily life, including recognizing individual hydration needs and adjusting fluid intake according to physical activity or environmental conditions [21,22,23,24,25,26,27]. Distinguishing between these dimensions is important, as factual knowledge alone may be insufficient to support adequate hydration behaviors without the capacity to translate knowledge into practice [28,29,30].

Perceptions of drinking water quality, particularly trust in tap water, have also emerged as important determinants of water consumption patterns. Public concern regarding tap water safety persists in many countries, including those with regulated municipal water supplies, and may influence water source choices and overall intake [31,32,33]. Higher trust in tap water quality has been associated with greater water consumption and reduced reliance on bottled water [34,35,36,37]. Sociodemographic factors, including education level, age, and gender, further contribute to variability in hydration knowledge, perceptions, and behaviors, although findings remain inconsistent across populations [17,23,31,38].

Although previous studies have examined hydration knowledge, water intake, and perceptions of drinking water quality, these factors are often investigated separately, and evidence from Eastern European populations remains limited. Given the central role of adequate hydration in renal health and the prevention and management of chronic kidney disease, understanding modifiable determinants of hydration behaviors has important clinical and public health relevance. Therefore, the present study aimed to assess hydration knowledge, water consumption patterns, and attitudes toward drinking water quality among adults residing in Romania, with particular emphasis on Western Romania.

## 2. Materials and Methods

### 2.1. Study Design and Setting

This study employed a cross-sectional quantitative survey design to assess hydration knowledge, water consumption patterns, and attitudes toward drinking water quality among adults in Romania, with a particular focus on Western Romania. The study was observational and non-interventional, with no manipulation of variables. All variables were measured at a single time point during the routine clinic visit; no intervention or exposure manipulation was performed.

Data were collected between November 2024 and November 2025 using a self-developed, paper-based questionnaire administered in person. Data collection was conducted continuously over this 12-month period; seasonality was not explicitly controlled for, as the study aimed to assess general hydration knowledge, perceptions, and self-reported water consumption behaviors rather than seasonal variation. The survey was conducted in four primary care clinics in Arad, Romania, where adult patients attending routine medical consultations were invited to participate. Primary care clinics were selected to facilitate pragmatic recruitment of adults during routine medical visits, reflecting real-world healthcare utilization.

The questionnaire was completed anonymously in a supervised clinical setting, without time constraints. Participation was voluntary and uncompensated. The study design aimed to capture both objective indicators (e.g., hydration knowledge and self-reported daily water intake) and subjective perceptions (e.g., trust in drinking water quality and safety).

### 2.2. Participants

A total of 165 adults were included in the final analysis. Participants were recruited using a convenience sampling approach from four primary care clinics in Arad, Romania. Eligible individuals were approached in person by a member of the research team during routine clinic visits and invited to participate.

No formal a priori sample size calculation was conducted. The sample size was determined by logistical feasibility and participant availability during the data collection period, using a convenience sampling approach in four primary care clinics.

Participants were eligible for inclusion if they met the predefined inclusion criteria and did not meet any exclusion criteria. These criteria, along with their rationale, are summarized in Table 1.

**Figure 1:**
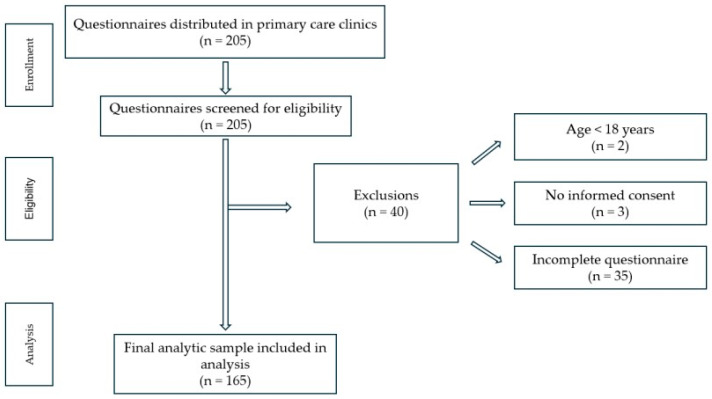
STROBE-style flow diagram of participant screening, exclusions, and final analytic sample.

After applying these criteria, a total of 165 participants with complete datasets were retained for statistical analysis. The participant screening and selection process is illustrated in a STROBE-style flow diagram (Figure 1).

### 2.3. Study Procedure

The study procedures were conducted in a standardized, stepwise manner. First, adult patients attending routine consultations at four primary care clinics in Arad, Romania, were approached in person by a member of the research team and informed about the purpose of the study. Individuals who expressed interest in participation were provided with detailed information regarding the study procedures and data confidentiality.

Second, written informed consent was obtained from all participants prior to data collection. Only individuals who provided consent and met the inclusion criteria were enrolled in the study.

Third, participants completed a structured, self-developed, paper-based questionnaire administered in a supervised clinical setting. The questionnaire was completed during the clinic visit, without time constraints, and no assistance was provided with item content to avoid response bias. Completed questionnaires were reviewed on-site for completeness.

Fourth, questionnaire responses were anonymized and manually entered into an electronic database. Data entry was performed using IBM SPSS Statistics (Version 28.0; IBM Corp., Armonk, NY, USA). A random subset of questionnaires was double-checked against the electronic database to minimize data entry errors.

Finally, statistical analyses were conducted according to a predefined analysis plan, including descriptive statistics, bivariate analyses, and multivariable regression models to examine associations between hydration knowledge, water consumption behaviors, and attitudes toward drinking water quality.

### 2.4. Survey Instrument

Data were collected using a structured, self-developed, paper-based questionnaire designed specifically for the purposes of this study. The questionnaire was developed to assess hydration knowledge, water consumption behaviors, and attitudes toward drinking water quality among adults. Details on questionnaire scoring and index construction are provided in Appendix A.

The questionnaire was designed to capture key domains relevant to hydration behavior in adult populations, based on constructs commonly assessed in prior hydration- and nutrition-related research. Item selection was informed by previously published instruments and conceptual frameworks evaluating hydration knowledge, attitudes, and water consumption behaviors. These frameworks commonly include domains related to recognition of dehydration symptoms, understanding of the physiological roles of water, awareness of recommended daily water intake, and knowledge of factors influencing individual fluid requirements, such as physical activity level and environmental conditions. Similar domain-based approaches have been applied in earlier studies examining hydration knowledge and related behaviors in adult populations.

The attitudinal items were designed to reflect constructs frequently reported in the literature on drinking water perception and choice, including trust in tap water quality, perceived safety of bottled water relative to tap water, and the importance attributed to water composition and filtration. All items were adapted to the Romanian context to ensure relevance to local drinking water practices and commonly expressed public concerns. Although the questionnaire was self-developed, its content was grounded in established theoretical and empirical literature addressing hydration-related knowledge, attitudes, and perceptions of drinking water quality.

Prior to data collection, the questionnaire items were reviewed internally by the research team to assess clarity, face validity, and relevance to the Romanian adult population. No formal expert panel evaluation or cognitive pre-testing was conducted. In addition, no formal construct validation or test–retest reliability assessment was performed, as the questionnaire was intended for exploratory use.

The questionnaire comprised four main content domains. First, sociodemographic characteristics were collected, including age, gender, residential environment (urban/rural), highest level of education attained, and employment status. These variables were used to describe the study population and to explore potential associations with hydration knowledge, water consumption behaviors, and attitudes toward drinking water quality.

Hydration knowledge was assessed using seven structured items covering key conceptual domains related to water intake and hydration. These domains included recognition of common symptoms of dehydration, understanding of the physiological roles of water in the human body, awareness of recommended daily water intake, and knowledge of factors influencing individual fluid requirements (e.g., physical activity level and environmental conditions). Each item had one correct response; correct answers were assigned a score of 1, while incorrect or “do not know” responses were assigned a score of 0. A total hydration knowledge score was calculated by summing individual item scores, with higher scores indicating greater hydration-related knowledge. For analytical purposes, hydration knowledge scores were further categorized into low, moderate, and high levels based on score distribution, as described in Section 2.6.

Water consumption behaviors were assessed using items addressing the primary type and source of drinking water, preferred packaging type (e.g., glass or plastic), self-reported daily water intake assessed using predefined intake categories (<1, 1–2, 2–3, and >3 L/day), preferred drinking water temperature, and frequency of consumption of specific water types, including carbonated and alkaline water. These items were primarily categorical and were used to characterize consumption patterns and to examine associations with hydration knowledge and attitudinal measures.

Attitudes toward drinking water quality and safety were evaluated using five Likert-scale statements addressing perceptions of water safety, trust, and decision-making related to water consumption. These included perceptions of trust in tap water quality, beliefs regarding the safety of bottled water compared with tap water, the importance attributed to knowing water composition, and the perceived necessity of filtering tap water. Responses were recorded on a 5-point Likert scale ranging from 1 (“strongly disagree”) to 5 (“strongly agree”), with higher scores indicating stronger agreement. The internal consistency of the attitudinal scale was assessed using Cronbach’s alpha.

A trust–perception index was constructed to summarize participants’ overall attitudes toward drinking water quality and safety. The index comprised Likert-scale items assessing trust in tap water quality, belief that bottled water is safer than tap water, perceived necessity of filtering tap water, and the importance attributed to knowing the chemical composition of drinking water. Item scores were coded in a consistent direction and summed to generate a composite score, with higher values indicating greater concern regarding water safety and lower trust in tap water. The index was analyzed as a continuous variable in regression analyses. Exploratory or confirmatory factor analysis was not performed, as the index was intended as a theory-informed composite measure for use in an exploratory study. The possible total score ranged from 4 to 20.

### 2.5. Data Preprocessing and Handling of Missing Data

Prior to statistical analysis, data were processed using a predefined, stepwise preprocessing pipeline to ensure data quality and reproducibility.

- All returned questionnaires were screened for eligibility (*n* = 205).
- Records were excluded if participants were younger than 18 years (*n* = 2) or did not provide informed consent (*n* = 3).
- Questionnaires with incomplete responses on key variables required for the planned analyses were excluded (*n* = 35).
- The final analytic dataset included complete cases only (*n* = 165).

No data imputation procedures were applied, as the primary analyses were conducted on complete datasets. The number of excluded records at each preprocessing step corresponds to the participant flow illustrated in the STROBE-style flow diagram (Figure 1).

### 2.6. Statistical Analysis

Statistical analyses were performed using IBM SPSS Statistics, Version 28.0 (IBM Corp., Armonk, NY, USA). Descriptive statistics were used to summarize sociodemographic characteristics, hydration knowledge scores, water consumption patterns, and attitudinal responses. Categorical variables were expressed as frequencies and percentages, while continuous variables were summarized using means and standard deviations.

Hydration knowledge scores were categorized into low, moderate, and high levels based on the distribution of total scores. Chi-square (χ^2^) tests were used to examine associations between hydration knowledge categories and selected sociodemographic variables, including age group, gender, and education level.

Associations between hydration knowledge, attitudes toward water quality, and water consumption behaviors were assessed using correlation analyses. Correlation coefficients were calculated to evaluate relationships between continuous and ordinal variables, as appropriate.

To identify independent predictors of daily water intake, a multivariable linear regression analysis was conducted, including age, gender, education level, hydration knowledge score, and the trust–perception index as independent variables. Model fit was assessed using the coefficient of determination (R^2^) and the F-statistic.

Multivariable logistic regression analyses were performed to identify predictors of binary outcomes, including bottled water being reported as the primary drinking water source and adequate daily water intake (≥2 L/day). Results are presented as odds ratios (ORs) with 95% confidence intervals (CIs).

For analyses examining hydration adequacy, daily water intake was dichotomized into inadequate (<2 L/day) and adequate (≥2 L/day). The ≥2 L/day cutoff was applied uniformly across participants as a pragmatic and commonly used benchmark in adult population studies, enabling comparability and statistical modeling in this exploratory analysis. This threshold was not intended to represent individualized hydration requirements, which may vary according to sex, body mass, physical activity level, and environmental conditions.

The internal consistency of Likert-scale items assessing attitudes toward water quality was evaluated using Cronbach’s alpha. All statistical tests were two-tailed, and a *p*-value < 0.05 was considered statistically significant.

Overall, statistical methods were selected based on the measurement level of the variables and the cross-sectional study design.

Given the exploratory nature of this cross-sectional study and the interrelated nature of the examined variables, formal adjustments for multiple comparisons were not applied. The analyses were designed to be hypothesis-generating rather than confirmatory, and controlling for multiple testing using highly conservative procedures (e.g., Bonferroni correction) could have increased the risk of Type II error and obscured potentially meaningful associations. Accordingly, results are interpreted with caution, and emphasis is placed on effect sizes and 95% confidence intervals rather than sole reliance on *p*-value thresholds. Findings should be considered exploratory and warrant confirmation in future studies using pre-specified hypotheses and larger samples.

### 2.7. Model Diagnostics

Multicollinearity among independent variables included in the multivariable regression models was assessed using variance inflation factors (VIFs) and tolerance statistics. All predictors demonstrated acceptable collinearity diagnostics (VIF < 2.0; tolerance > 0.20), indicating no evidence of problematic multicollinearity.

Model assumptions and fit were evaluated using residual inspection and goodness-of-fit statistics appropriate to the respective linear and logistic regression models. No violations of model assumptions were detected.

Sensitivity analyses were not performed given the exploratory nature of the study and the use of complete-case analyses; however, results were robust across multivariable models, including the same core set of predictors.

### 2.8. Ethical Considerations

The study was conducted in accordance with the principles of the Declaration of Helsinki. Ethical approval was obtained from the participating clinical practices: CMÎR Izvorul Sănătății (Approval No. 481/1/30 September 2024), Luchin Bojita S.R.L. (Approval No. 583/a/8 October 2024), Dr. Toth Praxis S.R.L. (Approval No. 43/1/11 October 2024), and Medical Service Centrum S.R.L. (Approval No. 189/2/11 October 2024).

Participation was voluntary, and written informed consent was obtained from all participants prior to questionnaire completion. Participants received verbal and written information regarding the purpose of the study, the anonymous nature of data collection, and their right to decline or withdraw participation at any time without consequences.

No personally identifiable information was collected. Completed questionnaires were stored securely, and access to the data was restricted to the research team in accordance with applicable data protection regulations.

### 2.9. Hypotheses of the Study

Based on existing evidence regarding hydration behaviors, water consumption patterns, and perceptions of drinking water quality, the present cross-sectional study was designed to test the following a priori hypotheses among adults in Romania:

1. Higher hydration knowledge is positively associated with daily water intake and with a greater likelihood of achieving adequate daily water intake (≥2 L/day).
2. Greater trust in tap water quality is positively associated with daily water intake, whereas greater concern regarding water safety is associated with increased reliance on bottled water as the primary drinking water source.
3. Sociodemographic factors, particularly education level, are significantly associated with hydration knowledge, water consumption behaviors, and attitudes toward drinking water quality.
4. Attitudes toward water quality, including the perceived importance of water composition and safety, are associated with specific consumption behaviors, such as checking mineral content information and choosing bottled water over tap water.

## 3. Results

### 3.1. Participant Characteristics

Table 2 summarizes the sociodemographic characteristics of the study population. A total of 165 adults completed the survey and were included in the final analysis. The sample was predominantly female, largely composed of adults aged 26–50 years, and mainly comprised urban residents. Most participants reported at least a bachelor’s degree, and employment was primarily within the private and public sectors.

### 3.2. Hydration Knowledge Scores

Hydration knowledge was assessed using seven structured items addressing dehydration symptoms, physiological roles of water, recommended daily intake, and determinants of fluid requirements. Overall, participants exhibited moderate levels of hydration knowledge, with higher recognition of dehydration symptoms and physiological functions of water compared with knowledge of recommended intake values.

When categorized into low, moderate, and high knowledge groups, significant associations were observed between hydration knowledge and education level as well as gender, whereas age group was not significantly associated with hydration knowledge (Table 3).

### 3.3. Water Consumption Patterns

#### 3.3.1. Type and Source of Drinking Water

As shown in Table 4, participants’ preferred types and sources of drinking water are summarized. The majority of respondents reported still bottled water as their primary drinking water source. Carbonated water, spring water, and natural mineral water were reported less frequently. With respect to packaging, glass bottles were the most commonly preferred option, while a smaller proportion of participants preferred plastic packaging or reported no specific packaging preference.

#### 3.3.2. Daily Water Intake and Consumption Behaviors

Table 5 presents participants’ daily water intake and related consumption behaviors. Most respondents reported a daily water intake within the 1–3 L range, while a smaller proportion reported consumption below 1 L per day. Room-temperature water was the most commonly preferred drinking temperature.

Patterns of carbonated water consumption varied, ranging from rare to daily intake. Alkaline water had been consumed at least occasionally by a substantial proportion of participants, whereas a smaller subgroup reported being unfamiliar with this type of water.

### 3.4. Attitudes Toward Water Quality

Table 6 illustrates participants’ responses to Likert-scale items assessing attitudes toward water quality and safety. Overall, responses indicated moderate to high concern regarding drinking water quality. The highest level of agreement was observed for the statement “Filtering tap water is necessary for safe consumption”, whereas trust in tap water quality showed lower levels of agreement. Agreement with the statement that bottled water is safer than tap water was moderate to high.

### 3.5. Associations Between Knowledge, Attitudes, and Consumption Behaviors

Table 7 presents correlations between hydration knowledge, attitudinal variables, and water consumption behaviors. Hydration knowledge was positively correlated with daily water intake, indicating higher intake among participants with higher knowledge scores. Trust in tap water was also positively associated with daily water intake, whereas higher concern regarding water safety was associated with a greater reliance on bottled water.

In addition, participants who attributed greater importance to the chemical composition of drinking water were more likely to report checking mineral content labels. All correlation coefficients and corresponding significance levels are shown in Table 7.

### 3.6. Multivariable Regression Analyses

Table 8 and Table 9 present the results of the multivariable regression analyses examining predictors of daily water intake and bottled water consumption, respectively. In the multivariable linear regression model (Table 8), education level, hydration knowledge score, and the trust–perception index were independently associated with daily water intake (R^2^ = 0.212, *p* < 0.001). Hydration knowledge showed a positive association with daily water intake, whereas higher concern regarding water safety was inversely associated with intake.

In the logistic regression model (Table 9), lower trust in tap water and a stronger belief that bottled water is safer than tap water were identified as significant predictors of bottled water being reported as the primary drinking water source.

### 3.7. Predictors of Adequate Daily Water Intake

Daily water intake was dichotomized into inadequate (<2 L/day) and adequate (≥2 L/day). A multivariable logistic regression analysis was conducted to identify independent predictors of adequate daily water intake (Table 10).

Higher hydration knowledge score was independently associated with increased odds of adequate daily water intake (OR = 1.28, 95% CI: 1.10–1.49, *p* = 0.002). Greater trust in tap water was also positively associated with adequate intake (OR = 1.31, 95% CI: 1.12–1.54, *p* < 0.001).

Education level remained a significant predictor of adequate daily water intake (OR = 1.35, 95% CI: 1.08–1.69, *p* = 0.009). In contrast, age and gender were not significantly associated with achieving adequate water intake. The overall model was statistically significant (χ^2^(5) = 24.86, *p* < 0.001).

**Figure 2:**
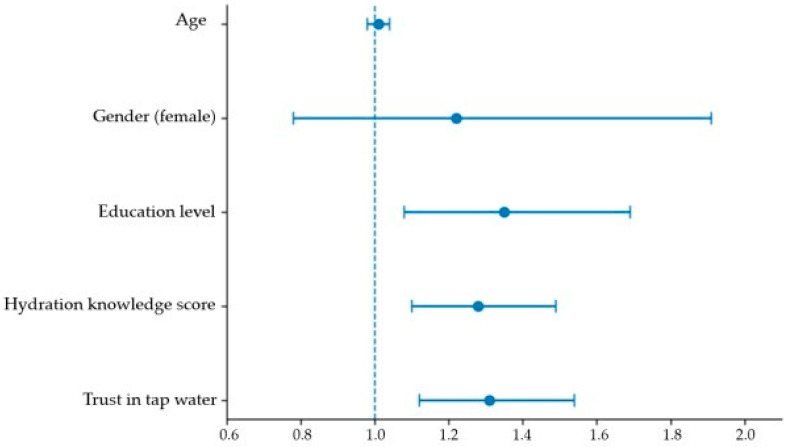
Forest plot of predictors of adequate daily water intake (≥2 L/day). Odds ratios (ORs) and 95% confidence intervals are shown for variables included in the multivariable logistic regression model (*n* = 165). Models were adjusted for age, gender, education level, hydration knowledge score, and trust in tap water. The vertical line indicates OR = 1. Statistical significance was defined as *p* < 0.05.

The independent predictors of adequate daily water intake identified in the multivariable model are visually summarized in Figure 2.

### 3.8. Reliability Analysis

The reliability analysis demonstrated acceptable internal consistency for the Likert-scale items assessing attitudes toward water quality, indicating that the questionnaire items consistently measure a common underlying construct. The overall internal consistency, assessed using Cronbach’s alpha, is presented in Table 11.

Item-level reliability analysis showed that removal of any single item resulted in only minimal changes in Cronbach’s alpha, suggesting that all items contribute meaningfully to the overall reliability of the scale (Table 12).

## 4. Discussion

The present study investigated hydration knowledge, water consumption behaviors, and attitudes toward drinking water quality in an adult population and identified several significant associations between these factors. Due to the cross-sectional design, the observed findings reflect associations rather than causal relationships. Overall, the findings support the study hypotheses and provide insight into modifiable determinants of hydration-related behaviors, while remaining exploratory in nature.

### 4.1. Hydration Knowledge and Water Intake

Higher hydration knowledge was independently associated with higher odds of achieving adequate daily water intake (≥2 L/day). These findings are consistent with previous studies demonstrating that individuals with better knowledge of hydration needs and the physiological roles of water are more likely to meet recommended fluid intake levels [39,40,41,42,43]. Similar associations between nutrition-related knowledge and healthier behavioral patterns have been reported across diverse populations [44,45,46,47]. Alternative explanations should also be considered, including the possibility of reverse causality, whereby individuals who habitually consume more water may acquire greater hydration-related knowledge over time, as well as residual confounding by unmeasured lifestyle or health-related factors.

However, not all studies have reported consistent associations, and some investigations have found weaker or non-significant relationships between hydration or nutrition-related knowledge and actual fluid intake. Such divergent findings suggest that knowledge alone may be insufficient to drive sustained behavior change and that contextual factors—such as access to drinking water, perceptions of water quality, habitual behaviors, and sociodemographic characteristics—may moderate this relationship [33,48,49].

The observed effect size, although modest, may be clinically relevant, as even small increases in daily fluid intake may contribute to improved hydration status and reduced risk of dehydration-related conditions, particularly among adults and vulnerable populations. These results highlight hydration knowledge as a potentially modifiable factor that may warrant further investigation in longitudinal and interventional studies [29,50,51].

### 4.2. Trust in Tap Water and Water Source Preferences

Greater trust in tap water quality was positively associated with daily water intake, while lower trust was associated with increased reliance on bottled water as the primary drinking water source. These findings align with prior research showing that perceptions of tap water safety strongly influence drinking behaviors and water source choices [23,37,52,53].

Nevertheless, evidence across studies is not entirely consistent, with some investigations reporting weaker or context-dependent associations between tap water perceptions and overall water intake. Such variability may reflect differences in local water infrastructure, regulatory environments, cultural norms, and the availability of alternative water sources.

Public concern regarding tap water quality has been widely documented, often driven by media coverage, past contamination events, and limited transparency regarding water treatment processes. Reduced trust in tap water may be associated with lower levels of adequate hydration, particularly in settings where bottled water is less accessible or affordable, thereby potentially exacerbating hydration disparities [32,54,55,56,57]. From a clinical perspective, addressing misconceptions about tap water safety may represent an important component of hydration counseling.

### 4.3. Sociodemographic Influences on Hydration Behaviors

Education level emerged as a consistent predictor across multiple outcomes, including hydration knowledge, daily water intake, and the likelihood of achieving adequate hydration. These findings are in agreement with existing literature, indicating that higher educational attainment is associated with greater health literacy and healthier lifestyle behaviors [58,59,60,61,62].

In contrast, age and gender were not independently associated with adequate daily water intake in multivariable analyses. While previous studies have reported mixed findings regarding demographic influences on hydration behaviors, the present results suggest that knowledge and attitudinal factors may play a more prominent role than basic demographic characteristics within this population [20,63,64].

### 4.4. Attitudes Toward Water Quality and Consumption Behaviors

Attitudes toward drinking water quality emerged as an important factor shaping water consumption behaviors. In particular, greater emphasis on water composition and perceived safety was associated with behaviors such as checking mineral content labels and preferring bottled water as the primary drinking water source. These findings are consistent with earlier studies indicating that individuals who place greater importance on water quality attributes are more likely to engage in selective and information-driven water consumption practices [23,37,65].

While such behaviors may reflect health consciousness, increased bottled water consumption has been associated in prior research with economic and environmental implications. Clinicians and public health professionals should therefore balance the promotion of informed water choices with evidence-based guidance on safe and sustainable hydration practices [36,66,67,68].

### 4.5. Implications for Clinical Practice and Public Health

The findings suggest that hydration behaviors are influenced by a dynamic interplay of hydration knowledge, perceptions of drinking water quality, and sociodemographic factors, with direct implications for clinical practice—particularly in primary care settings. Inadequate hydration represents a frequently overlooked but modifiable risk factor that may contribute to nonspecific symptoms such as fatigue, headaches, impaired concentration, and reduced overall well-being in adult patients. The results suggest that brief, targeted hydration counseling integrated into routine clinical encounters may be associated with healthier hydration behaviors; however, causal effects cannot be inferred from the present study.

Because higher hydration knowledge was independently associated with greater daily water intake and adequate hydration, the incorporation of simple hydration knowledge screening into routine consultations may be considered a supportive strategy, particularly for patients presenting with symptoms that may be related to insufficient fluid intake. Short educational interventions addressing recommended daily fluid intake, early signs of dehydration, and individual factors influencing hydration requirements (e.g., physical activity level and environmental conditions) could be delivered efficiently within standard appointment timeframes.

The observed association between trust in tap water and both daily water intake and hydration adequacy highlights the clinical importance of addressing patient perceptions regarding drinking water safety. Misconceptions regarding drinking water quality may have clinically important implications for populations with chronic kidney disease or cardiometabolic risk. Individuals in these groups are often advised to maintain adequate hydration to support renal function, blood pressure regulation, and metabolic control. However, concerns about tap water safety may lead to reduced overall fluid intake or increased reliance on bottled water, which can be costly, less accessible, and in some cases higher in sodium or mineral content. Such behaviors may inadvertently compromise hydration adequacy and dietary management in vulnerable populations, underscoring the importance of addressing water quality perceptions as part of routine clinical counseling.

Given that participants were recruited from primary care clinics, the findings may be relevant to routine clinical practice. Primary care providers are well positioned to identify individuals at risk of inadequate hydration and to deliver brief, personalized counseling tailored to patients’ educational background, hydration knowledge, and attitudes toward drinking water quality. Such opportunistic interventions may be particularly relevant for individuals with chronic conditions, older adults, and patients with lower health literacy, for whom adequate hydration supports symptom management and overall health maintenance.

At the public health level, the findings underscore the importance of transparent communication regarding drinking water quality and safety, alongside population-level educational initiatives aimed at improving hydration knowledge. Public health strategies that enhance trust in municipal water supplies and promote evidence-based hydration recommendations may contribute to improved hydration behaviors, while sustainability and cost considerations related to bottled versus tap water consumption should be understood in the broader public health context. Coordinated efforts between healthcare systems and public health authorities may therefore maximize both individual- and population-level benefits related to hydration. These clinical and public health implications should therefore be interpreted cautiously and viewed as hypothesis-generating rather than prescriptive.

### 4.6. Limitations of the Study

Several limitations of this study should be acknowledged. First, the cross-sectional design precludes causal inference; therefore, the observed associations should be interpreted with caution. Second, water intake and consumption behaviors were self-reported, which may have introduced recall bias or social desirability bias.

Hydration knowledge and attitudes were assessed using a self-developed questionnaire that did not undergo formal pilot testing, construct validation, criterion validation, or test–retest reliability assessment prior to data collection. Although the instrument was informed by existing literature and internally reviewed for clarity and face validity, limitations in psychometric validation may affect measurement precision. In addition, hydration knowledge categories were defined using score distribution rather than externally validated thresholds, which may limit comparability with other studies. Some attitudinal items demonstrated high mean scores, suggesting strong consensus but also raising the possibility of ceiling effects that may have limited variability.

The study sample was relatively small and recruited using a convenience sampling approach from adults attending primary care clinics in Western Romania, predominantly in urban settings, which may limit generalizability to other populations or contexts. No formal a priori sample size or power calculation was performed; although the final sample size was sufficient to detect several statistically significant associations, the study may have been underpowered to identify smaller effect sizes or subgroup differences.

Hydration status was assessed using self-reported daily water intake rather than objective biomarkers such as urine osmolality or validated hydration indices. Water intake was recorded using predefined intake categories rather than as a continuous measure, which may have limited sensitivity and contributed to clustering of responses. Adequate hydration was defined using a uniform cutoff of ≥2 L/day for all participants; while commonly applied in epidemiological research, this threshold does not account for individual variability related to sex, body mass, physical activity level, or environmental conditions, and some degree of misclassification cannot be excluded. In addition, data collection occurred over a 12-month period without explicit control for seasonality, which may have influenced self-reported water intake and hydration perceptions.

Finally, although multiple relevant covariates were included in the analyses, residual confounding by unmeasured variables cannot be excluded. Multiple statistical tests were conducted without formal adjustment for multiple comparisons; therefore, findings should be interpreted as exploratory and hypothesis-generating rather than confirmatory.

Despite these limitations, the study provides valuable insight into modifiable determinants of hydration behaviors in an understudied population and offers a foundation for future longitudinal and interventional research.

### 4.7. Future Research Directions

Future research should investigate longitudinal relationships between hydration knowledge, perceptions of drinking water quality, and hydration behaviors to better clarify potential causal pathways. The inclusion of objective hydration markers, such as urine osmolality or validated hydration indices, would strengthen the assessment of hydration status and complement self-reported intake measures.

In addition, intervention studies are needed to evaluate the effectiveness of targeted educational and communication strategies aimed at improving hydration knowledge and addressing concerns regarding tap water safety. Such interventions may be particularly relevant in clinical and community settings, where healthcare professionals play a key role in promoting healthy hydration behaviors. Further research across diverse populations and geographic contexts would also enhance the generalizability of findings and inform evidence-based hydration recommendations.

From a theoretical perspective, the present findings contribute to the growing body of evidence conceptualizing hydration behavior as a multidimensional phenomenon influenced by knowledge, attitudes, and contextual perceptions rather than physiological needs alone. The integrated examination of hydration knowledge and drinking water quality perceptions supports behavioral and health literacy frameworks that emphasize the interaction between cognitive and environmental determinants of health behaviors. From a practical standpoint, the results suggest potential applications in primary care and public health settings, including the development of brief hydration knowledge screening tools, targeted educational interventions, and communication strategies aimed at improving trust in tap water quality. Future research should build on these findings through longitudinal and interventional studies to assess causal pathways and to evaluate the effectiveness of knowledge- and perception-based interventions in improving hydration behaviors and health outcomes.

## 5. Conclusions

This study highlights the important roles of hydration knowledge and perceptions of drinking water quality in shaping water consumption behaviors among adults. Higher hydration knowledge and greater trust in tap water were independently associated with higher daily water intake and a greater likelihood of achieving adequate hydration.

These findings suggest that modifiable factors, such as knowledge and attitudes toward water quality, are meaningfully associated with hydration behaviors and may be relevant considerations for clinical counseling and public health interventions. Enhancing hydration education and promoting transparent communication regarding drinking water safety may be associated with healthier and more sustainable water consumption practices, within the broader context of sustainability considerations discussed in the literature.

From a clinical perspective, these findings support the potential value of incorporating brief hydration knowledge screening into routine primary care encounters, particularly for patients at risk of inadequate hydration. In addition, providing clear, evidence-based education regarding local drinking water quality and tap water safety may help address misconceptions that act as barriers to adequate hydration.

Further research using longitudinal designs and objective hydration measures is warranted to confirm these associations and to evaluate the effectiveness of targeted educational and communication strategies in improving hydration outcomes. Because this study was cross-sectional, these findings should be interpreted as associative and hypothesis-generating, and confirmation in longitudinal and interventional studies is needed.

## Figures and Tables

**Table 1:** Inclusion and exclusion criteria and rationale.

Inclusion Criteria	Exclusion Criteria	Rationale
Age ≥ 18 years	Age < 18 years	To restrict the sample to an adult population
Provision of informed consent prior to participation	Failure to provide informed consent	Ethical requirement for participation
Completion of all mandatory questionnaire items	Incomplete questionnaires or missing responses on key variables	To ensure data completeness and valid statistical analysis
Residence in Romania	Residence outside Romania	To ensure relevance to the target population
Ability to read and understand Romanian	Inability to read or understand Romanian	To ensure accurate comprehension of questionnaire items

**Table 2:** Sociodemographic characteristics of the study population (*n* = 165).

Variable	Category	*n*	%
Gender	Female	115	69.7
	Male	50	30.3
Age group	18–25 years	34	20.6
	26–35 years	51	30.9
	36–50 years	66	40.0
	>50 years	14	8.5
Residential environment	Urban	116	70.3
	Rural	49	29.7
Education level	High school	53	32.1
	Bachelor’s degree	66	40.0
	Master’s degree	39	23.6
	Doctorate	7	4.3
Employment status	Private sector	64	38.8
	Public sector	53	32.1
	Unemployed	31	18.8
	Self-employed	17	10.3

**Table 3:** Associations between hydration knowledge category and demographic variables (*n* = 165).

Demographic Variable	χ^2^	df	*p*-Value
Education level	17.89	6	0.006
Gender	20.25	2	<0.001
Age group	6.80	6	0.339

**Table 4:** Preferred types and sources of drinking water and packaging preferences (*n* = 165).

Variable	Category	*n*	%
Most frequently consumed water type	Still bottled water	128	77.6
	Carbonated water	15	9.1
	Spring water	13	7.9
	Natural mineral water	7	4.2
Preferred packaging	Glass	89	53.9
	Plastic	23	13.9
	No preference	44	26.7
	Other	9	5.5

**Table 5:** Daily water intake and consumption behaviors among participants (*n* = 165).

Variable	Category	*n*	%
Daily water intake	<1 L/day	17	10.3
	1–2 L/day	87	52.7
	2–3 L/day	57	34.5
	>3 L/day	4	2.4
Preferred water temperature	Room temperature	117	70.9
	Cold	33	20.0
	No preference	10	6.1
	Lukewarm	5	3.0
Carbonated water consumption	Rarely	83	50.3
	Several times/week	37	22.4
	Daily	17	10.3
	Never	28	17.0
Tried alkaline water	Yes, occasionally	98	59.4
	Yes, frequently	11	6.7
	Never	31	18.8
	Unfamiliar	24	14.5

**Table 6:** Descriptive statistics of Likert-scale items assessing attitudes toward water quality (*n* = 165).

Likert-Scale Statement	Mean
It is important to know the chemical composition of the water I consume	3.82
I trust the quality of tap water	2.85
I choose water based on recommendations from nutrition specialists	2.90
Bottled water is safer than tap water	3.70
Filtering tap water is necessary for safe consumption	4.01

**Table 7:** Correlations among hydration knowledge, attitudes toward water quality, and consumption behaviors (*n* = 165).

Variable Pair	Correlation Coefficient (r)	*p*-Value
Hydration knowledge ↔ Daily water intake	0.21	0.006
Trust in tap water ↔ Daily water intake	0.27	0.001
Hydration knowledge ↔ Trust–perception index	−0.19	0.012
Importance of water composition ↔ Checking mineral content	0.30	<0.001

Note: The arrow (↔) indicates the direction of the correlation between paired variables.

**Table 8:** Multiple linear regression model for daily water intake (*n* = 165).

Predictor	Standardized β	t	*p*-Value
Age	−0.08	−1.06	0.291
Gender	0.11	1.52	0.130
Education level	0.17	2.39	0.018
Hydration knowledge score	0.23	3.11	0.002
Trust–perception index	−0.19	−2.60	0.010

Note: Model fit: R^2^ = 0.212; F(5, 159) = 8.54; *p* < 0.001.

**Table 9:** Logistic regression model for bottled water as the primary drinking water source (*n* = 165).

Predictor	Odds Ratio (OR)	95% CI	*p*-Value
Age	1.02	0.98–1.07	0.290
Education level	1.24	1.03–1.51	0.027
Trust in tap water	0.72	0.58–0.89	0.003
Belief that bottled water is safer	1.51	1.22–1.91	<0.001
Checking expiration date	1.43	1.10–1.87	0.008

Note: Model fit: χ^2^ = 18.92; *p* < 0.001.

**Table 10:** Logistic regression model for adequate daily water intake (≥2 L/day).

Predictor	OR	95% CI	*p*-Value
Age	1.01	0.98–1.04	0.290
Gender (female)	1.22	0.78–1.91	0.381
Education level	1.35	1.08–1.69	0.009
Hydration knowledge score	1.28	1.10–1.49	0.002
Trust in tap water	1.31	1.12–1.54	<0.001

Note: Adequate daily water intake defined as ≥2 L/day. Model fit: χ^2^(5) = 24.86, *p* < 0.001.

**Table 11:** Overall scale reliability statistics for attitudes toward water quality (*n* = 165).

Estimate	Cronbach’s α
Point estimate	0.78
95% CI lower bound	0.74
95% CI upper bound	0.79

**Table 12:** Individual item reliability statistics (Cronbach’s α if item deleted).

Statement	Cronbach’s α If Item Deleted
Importance of knowing water composition	0.76
Trust in tap water quality	0.79
Choosing water based on nutritionist recommendations	0.77
Bottled water is safer than tap water	0.75
Necessity of filtering tap water	0.74

## Data Availability

The data presented in this study are available on reasonable request from the corresponding author. The data are not publicly available due to ethical considerations related to the protection of participant privacy.

## Abbreviations

The following abbreviations are used in this manuscript:

VIF	Variance Inflation Factor

## Appendix A. Questionnaire Scoring Overview

Hydration knowledge was assessed using seven items scored as 1 = correct and 0 = incorrect or “do not know,” yielding a total score range of 0–7, with higher scores indicating greater hydration knowledge. Example knowledge domains included recognition of dehydration symptoms and awareness of recommended daily water intake.

Attitudes toward drinking water quality were assessed using five items rated on a 5-point Likert scale (1 = strongly disagree to 5 = strongly agree).

A trust–perception index was calculated by summing four Likert-scale items after coding responses in a consistent direction, resulting in a total score range of 4–20, with higher scores indicating greater concern regarding drinking water safety and lower trust in tap water.
